# Robustness of radiomics features on 0.35 T magnetic resonance imaging for magnetic resonance-guided radiotherapy

**DOI:** 10.1016/j.phro.2024.100613

**Published:** 2024-07-20

**Authors:** Morgan Michalet, Gladis Valenzuela, Pierre Debuire, Olivier Riou, David Azria, Stéphanie Nougaret, Marion Tardieu

**Affiliations:** a Institut du Cancer de Montpellier, Fédération Universitaire d’Oncologie-Radiothérapie d’Occitanie Méditerranée (FOROM), INSERM U1194 IRCM, 208 avenue des apothicaires, 34298 Montpellier, France; b IRCM, Univ Montpellier, ICM, INSERM, 208 avenue des apothicaires, 34298 Montpellier, France; c Institut du Cancer de Montpellier, Service d’imagerie médicale, 208 avenue des apothicaires, 34298 Montpellier, France

**Keywords:** Radiomics, Robustness, MR-guided radiotherapy

## Abstract

**Background and purpose:**

MR-guided radiotherapy adds the precision of magnetic resonance imaging (MRI) to the therapeutic benefits of a linear accelerator. Prior to each therapeutic session, an MRI generates a significant volume of imaging data ripe for analysis. Radiomics stands at the forefront of medical imaging and oncology research, dedicated to mining quantitative imaging attributes to forge predictive models. However, the robustness of these models is often challenged.

**Materials and methods:**

To assess the robustness of feature extraction, we conducted reproducibility studies using a 0.35 T MR-linac system, employing both a specialized phantom and patient-derived images, focusing on cases of pancreatic cancer. We extracted shape-based, first-order and textural features from patient-derived images and only first-order and textural features from phantom-derived images. The impact of the delay between simulation and first fraction images was also assessed with an equivalence test.

**Results:**

From 107 features evaluated, 58 (54 %) were considered as non-reproducible: 18 were uniformly inconsistent across both phantom and patient images, 9 were specific to phantom-based analysis, and 31 to patient-derived data.

**Conclusion:**

Our findings show that a significant proportion of radiomic features extracted from this dual dataset were unreliable. It is essential to discard these non-reproducible elements to refine and enhance radiomic model development, particularly for MR-guided radiotherapy in pancreatic cancer.

## Highlights

• Dimensionality reduction is mandatory for radiomics analyses.
• Test-retest studies allow for a robust dimensionality reduction.
• MR-linacs provide a large amount of data that can be used for radiomic analysis.
• A significant proportion (54 %) of radiomics features extracted from phantom and patient data from a 0.35 T MR-linac are non-reproducible.

## 1 Introduction

Magnetic Resonance-guided radiotherapy (MRgRT) represents a groundbreaking advancement in the management of cancer, integrating the diagnostic benefits of magnetic resonance imaging (MRI) to the therapeutic power of a linear accelerator. This innovative merger is embodied in the MR-linacs, which utilizes a 0.35 T or 1.5 T MRI scanner to offer unprecedented precision in cancer treatment [1]. The system’s design facilitates meticulous patient positioning through daily image acquisition and enables real-time tracking of the target with continuous cine-MRI during radiation delivery. This dynamic imaging allows for on-the-fly adjustments, pausing the radiation if the target drifts beyond the established safety margins, thereby optimizing treatment accuracy and protecting surrounding healthy tissue.

Radiomics has emerged as a transformative field in medical imaging and oncology, harnessing the power of advanced computing to extract a plethora of quantitative imaging features. These features are the building blocks for sophisticated algorithms that aim to correlate image data with biological characteristics and predict clinical outcome [2], [3]. Radiomics studies should comply with several steps in a typical workflow [4], [5]. One of these steps is to reduce the number of extracted features by removing those that are not robust or those that are redundant for the analysis. This approach, known as dimensionality reduction, reduces the risk of overfitting during model training [6]. This step is crucial for the integrity and applicability of the predictive models generated through radiomic analysis.

In the context of MR-linacs, the large volume of imaging data generated daily offers a fertile ground for radiomic research. The images could also be used to perform delta-radiomics analysis, which represents a comparative longitudinal analysis that evaluates how radiomic features evolve over the course of treatment, providing insights into the biological effects of radiotherapy on tumor and normal tissues.

The focus of this study is to assess the reproducibility of radiomic features derived from two sources: a specialized phantom, and actual patient images from those undergoing treatment for pancreatic cancer using the 0.35 T MRI scanner of an MR-linac system. By examining the consistency of these features, we aim to identify which can be reliably used to enhance the personalization and efficacy of MR-guided radiotherapy. The implications of this research are vast, with the potential to significantly refine radiomic models and ultimately, propel the evolution of patient-specific cancer therapy.

## 2 Materials and methods

**Fig. 1:**
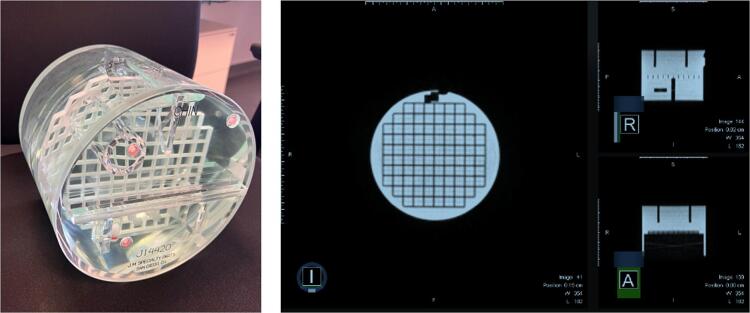
ACR phantom and images of this phantom with a 0.35 T MRI.

We conducted a detailed investigation into the consistency of radiomic features derived from MR images using a standardized phantom, specifically the American College of Radiology (ACR) phantom, which includes a diverse array of geometric configurations (as displayed in Fig. 1). This phantom is routinely utilized for the calibration and quality assurance of MR imaging systems. In addition, we extended our analysis to a patient cohort undergoing treatment for pancreatic cancer, comparing imaging data from pre-treatment simulations to those obtained on the first day of therapy.

### 2.1 Patient criteria

Our study encompassed seventy-four patients treated either for borderline or locally advanced pancreatic cancer with Stereotactic MR-guided Adaptive Radiotherapy (SMART). Treatment regimens varied, with three patients receiving a total dose of 40 Gy across five sessions, and the remaining seventy-one receiving 50 Gy in the same number of fractions. Prior to the SMART regimen, all patients had been treated with induction chemotherapy. Crucially, no chemotherapy was administered in the interim between the simulation examination and the initial fraction of SMART treatment. Participation in this study was contingent upon informed consent, with the study's protocol being duly recorded in the Health Data Hub (registration number: #1802) and receiving the endorsement of the COMERE local research committee (ICM-ART 2020/01).

### 2.2 Image acquisition

Imaging for both the phantom and patient studies was conducted using the standard MRIdian® protocol for pancreatic cancer: a balanced steady-state gradient echo sequence (True FISP) with a flexible torso phased array coil and characterized by a resolution of 1.63 × 1.63 × 3 mm^3^, a 276 × 276 matrix size, a TR/TE of 3.84/1.62 ms, a flip angle of 60° and a rapid 17-second acquisition period. For the phantom, eleven sessions of five consecutive acquisitions were performed without repositioning the phantom over three weeks, culminating in a total of 55 acquisitions. The phantom was repositioned between each session. For patients, six MRI scans were performed: one for each of the five SMART fractions and an additional simulation scan conducted. This simulation scan was classically performed from 5 to 14 days after the last chemotherapy injection. To avoid motion artefacts, a breath-hold technique at physiological end-expiration was employed during the imaging process.

### 2.3 Image pre-processing

Prior to the feature extraction, images underwent a series of pre-processing steps designed to mitigate artefacts and enhance the reliability of subsequent feature calculations. These steps, executed using an in-house python code and adhering to IBSI guidelines [7], included bias field correction via the N4 algorithm [8], noise reduction through anisotropic diffusion filtering (settings: number of iterations = 5; kappa = 5; gamma = 3) [9], [10], and image B-spline interpolation to achieve isotropic voxel dimensions of 1.63×1.63×1.63 mm^3^.

### 2.4 Features extraction

The feature extraction was preceded by the application of a fixed bin width of 7 for the gray-level discretisation, performed using the open source software Pyradiomics v3.0.1 [11]. As all acquired images had a minimum and maximum value of [0 455] which can be considered like normalized images, this resulting in 65 bins. In total, 107 features were extracted from each image, spanning shape-based metrics, first-order and second-order statistics, as well as advanced textural features via Gray Level Co-occurrence Matrix (GLCM), Gray Level Size Zone Matrix (GLSZM), Gray Level Run Length Matrix (GLRLM), Neighbouring Gray Tone Difference Matrix (NGTDM), and Gray Level Dependence Matrix (GLDM). It is important to note that no filters were used on the images prior to the extraction of these features. Texture features were aggregated using the 3DAverage method (ITBB). For the phantom study, the region of interest (ROI) was automatically outlined using custom in-house code in Matlab and included the entire phantom. For the cohort of pancreatic cancer patients, feature extraction was performed on the gross tumor volume (GTV), which was identified by the treating radiation oncologist on the simulation MRI and then rigidly aligned with the MRI from the first fraction of treatment. If necessary, the radiation oncologist could adjust the GTV contour on the first fraction MRI.

Feature extraction commenced with the application of a consistent bin width of 7 for gray-level discretization, facilitated by the open-source software Pyradiomics v3.0.1. Given that all images were within a value range of [0, 455]—effectively normalized—this process resulted in the formation of 65 bins. Subsequently, 107 features were extracted from each image, encompassing shape-based metrics, first-order and second-order statistics, and advanced textural features. These textural features included analysis through Gray Level Co-occurrence Matrix (GLCM), Gray Level Size Zone Matrix (GLSZM), Gray Level Run Length Matrix (GLRLM), Neighbouring Gray Tone Difference Matrix (NGTDM), and Gray Level Dependence Matrix (GLDM). It's critical to mention that the images underwent no filtration prior to feature extraction. The texture features were compiled using the 3DAverage method (ITBB). For the phantom study, the region of interest (ROI) was precisely defined using bespoke Matlab code and encompassed the entire phantom. In the case of the pancreatic cancer patient cohort, feature extraction was conducted on the gross tumor volume (GTV), which was delineated by the treating radiation oncologist during the simulation MRI and subsequently aligned with the MRI from the initial treatment fraction.

### 2.5 Statistical analysis

In the phantom study, for each set of five consecutive acquisitions, we calculated both the mean and the coefficient of variation (CoV) for each feature. The CoV, defined as the standard deviation divided by the mean, served as a gauge for the repeatability of the findings. Since the phantom's ROI was automatically segmented, ensuring an identical ROI across all images, shape-based features were excluded from the repeatability assessment. The CoV for each feature was then calculated across all sessions based on each session's average, effectively measuring the phantom's reproducibility. Features were divided into three groups as excellent repeatability (CoV ≤ 5%), good repeatability (5 % < CoV ≤ 10 %) and poor repeatability (10 % < CoV).

For the patient cohort, reproducibility was evaluated by comparing features extracted from the simulation scan and the first treatment fraction. We calculated the intraclass correlation coefficient (ICC) for each feature using Python (version 3.8.18), incorporating the libraries Pandas (version 2.0.3) and Pingouin (version 0.5.4). The ICC is a statistical measure that determines the consistency of measurements by comparing the variance of the same subject to the total variance across all ratings and subjects. Features were subsequently categorized based on their ICC values: excellent reproducibility (ICC ≥ 0.90), good reproducibility (0.75 ≤ ICC < 0.90), and poor reproducibility (ICC ≤ 0.75). The influence of the delay between the simulation MRI and the first fraction MRI was assessed by comparing the mean variation of each feature between two groups of patients based on the median delay between the two MRI. In addition, equivalence tests were performed to evaluate the similarity of feature values between these groups. This comprehensive analysis is poised to contribute significantly to the precision and effectiveness of radiomic research, particularly in the context of MR-guided radiotherapy for pancreatic cancer.

## 3 Results

### 3.1 Analysis of phantom-derived radiomic features

For each imaging session of the ACR phantom, a comprehensive suite of 93 radiomic features was extracted from the acquired data. All these features are listed in supplementary material in Table S1. The Fig. 1 provides a representative image of the phantom used in this study as well as its image using 0.35 T MRI. Shape features were deliberately omitted from this phase of analysis due to the focus on other feature categories.

Upon evaluation, 27 of these features (accounting for 29 % of the total) were identified as having suboptimal reproducibility, evidenced by a coefficient of variation (CoV) exceeding 10 % in at least one session or across all sessions cumulatively. Conversely, a significant proportion of the features, 44 in total (47 %), demonstrated high repeatability with a CoV of less than 5 %. Furthermore, 22 features (24 %) displayed good repeatability, falling into the CoV range of greater than 5 % but less than 10 %. The features with poor repeatability are listed in Table 1. The features falling into other categories of repeatability—good, and high—are systematically catalogued in supplementary material in Tables S2 and S3.

**Table 1:** List of poorly-repeatable features on phantom images and their coefficient of variation (CoV > 10 %).

Feature	Coefficient of variation (CoV)
original_firstorder_Minimum	30.7 %
original_firstorder_Variance	10.1 %
original_glcm_Autocorrelation	10.8 %
original_glcm_ClusterProminence	21.0 %
original_glcm_ClusterTendency	11,4%
original_glcm_SumSquares	10.8 %
original_gldm_GrayLevelVariance	10.2 %
original_gldm_HighGrayLevelEmphasis	10.8 %
original_gldm_LargeDependenceLowGrayLevelEmphasis	15.7 %
original_gldm_LowGrayLevelEmphasis	18.5 %
original_gldm_SmallDependenceHighGrayLevelEmphasis	16.1 %
original_gldm_SmallDependenceLowGrayLevelEmphasis	18.4 %
original_glrlm_HighGrayLevelRunEmphasis	10.9 %
original_glrlm_LongRunLowGrayLevelEmphasis	18.6 %
original_glrlm_LowGrayLevelRunEmphasis	19.1 %
original_glrlm_ShortRunHighGrayLevelEmphasis	11.6 %
original_glrlm_ShortRunLowGrayLevelEmphasis	19.4 %
original_glszm_HighGrayLevelZoneEmphasis	12.1 %
original_glszm_LargeAreaEmphasis	10.8 %
original_glszm_LargeAreaLowGrayLevelEmphasis	20.3 %
original_glszm_LowGrayLevelZoneEmphasis	20.3 %
original_glszm_SmallAreaHighGrayLevelEmphasis	12.4 %
original_glszm_SmallAreaLowGrayLevelEmphasis	20.5 %
original_glszm_ZoneVariance	10.8 %
original_ngtdm_Complexity	15.8 %
original_ngtdm_Strength	11.1 %
original_glcm_ClusterShade	16.0 %

CoV = Coefficient of variation

To illustrate the concept of CoV and its application within our study, the Fig. S1 in supplementary material provides a visual comparison of this metric across four distinct radiomic features. This comparison underscores the variability and potential reliability of each feature in the context of repeated phantom imaging sessions.

### 3.2 Analysis of the patient-derived radiomic features

**Fig. 2:**
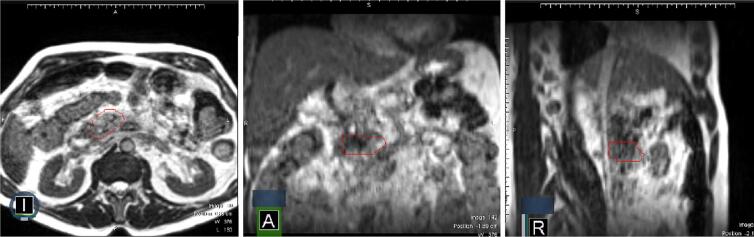
Example of patient images with 0.35 T MRI, GTV (ROI) delineated in red.

**Fig. 3:**
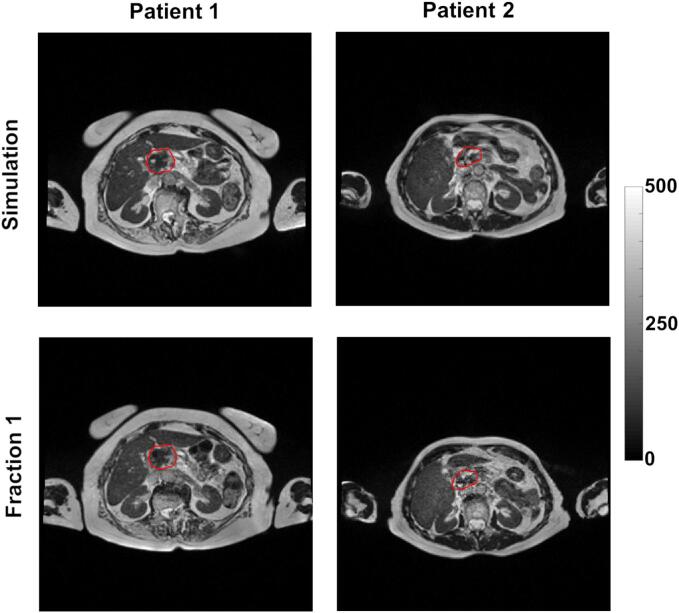
Example of comparison between the simulation and first fraction images for two different patients, after bias field and noise corrections; GTV in red.

In our analysis of patient-derived data, we meticulously extracted 107 distinct radiomic features from the gross tumor volume (GTV) delineated in each patient's MRI scan, with the full list of these features available in the Table S4 in supplementary material. The Fig. 2 provides a visual representation in 3 plans of a patient's MRI with the GTV clearly marked. The Fig. 3 provides comparison of simulation and first fraction images for two different patients. Out of these features, a significant number—49 features, which equates to 46 %—demonstrated poor reproducibility, as indicated by an intraclass correlation coefficient (ICC) falling below the 75 % threshold. Conversely, we identified 28 features (26 % of the total) that exhibited high reproducibility, with an ICC exceeding 90 %. The remaining 30 features (28 %) showed good reproducibility, with their ICCs ranging between 75 % and 90 %. The features with poor reproducibility are listed in Table 2. The features falling into other categories of reproducibility— good, and high—are systematically catalogued in supplementary material in Tables S5 and S6.

**Table 2:** List of poorly-reproducible GTV features on simulation vs first fraction images and their interclass correlation (ICC < 75 %).

Feature	Interclass Correlation (ICC)
original_firstorder_RootMeanSquared	31.6 %
original_glcm_JointAverage	27.9 %
original_glcm_Contrast	11.8 %
original_gldm_HighGrayLevelEmphasis	8.3 %
original_firstorder_Range	26.1 %
original_gldm_DependenceNonUniformityNormalized	60.9 %
original_glszm_LargeAreaLowGrayLevelEmphasis	45.9 %
original_glcm_DifferenceEntropy	66.7 %
original_gldm_LargeDependenceHighGrayLevelEmphasis	29.3 %
original_ngtdm_Strength	28.6 %
original_glszm_GrayLevelVariance	11.0 %
original_glcm_SumAverage	27.9 %
original_glszm_ZoneVariance	74.1 %
original_firstorder_InterquartileRange	51.6 %
original_firstorder_Maximum	23.3 %
original_firstorder_90Percentile	35.0 %
original_firstorder_10Percentile	39.9 %
original_firstorder_Variance	14.5 %
original_glcm_ClusterTendency	16.3 %
original_glszm_SmallAreaHighGrayLevelEmphasis	7.7 %
original_glcm_DifferenceVariance	9.8 %
original_glszm_LargeAreaEmphasis	74.1 %
original_glszm_SmallAreaEmphasis	58.5 %
original_gldm_DependenceVariance	69.3 %
original_glcm_SumSquares	15.2 %
original_firstorder_Skewness	73.3 %
original_glszm_ZonePercentage	73.5 %
original_glrlm_GrayLevelVariance	14.2 %
original_glrlm_ShortRunHighGrayLevelEmphasis	8.3 %
original_firstorder_RobustMeanAbsoluteDeviation	50.0 %
original_firstorder_Mean	31.5 %
original_firstorder_Energy	22.8 %
original_firstorder_Median	31.4 %
original_gldm_SmallDependenceHighGrayLevelEmphasis	5.8 %
original_glszm_HighGrayLevelZoneEmphasis	9.1 %
original_glrlm_HighGrayLevelRunEmphasis	8.4 %
original_gldm_GrayLevelVariance	14.5 %
original_glcm_Autocorrelation	8.0 %
original_ngtdm_Contrast	52.6 %
original_gldm_SmallDependenceEmphasis	69.3 %
original_ngtdm_Complexity	2.9 %%
original_glcm_ClusterProminence	1.3 %
original_firstorder_MeanAbsoluteDeviation	44.3 %
original_glcm_ClusterShade	6.0 %
original_glrlm_LongRunHighGrayLevelEmphasis	8.8 %
original_glcm_DifferenceAverage	37.9 %
original_glszm_SizeZoneNonUniformity	69.0 %
original_glszm_SizeZoneNonUniformityNormalized	54.3 %
original_firstorder_TotalEnergy	22.8 %

ICC=Interclass correlation

The median delay between the two MRI was 17 days (range 5–33). We did not find any differences of mean feature variation between the 2 groups for the different features, as indicated by the significant results of the equivalence tests conducted, with different ICC results tested. The Table S7 shows 4 examples of mean variation of features exhibiting low reproducibility and 4 examples of features exhibiting high reproducibility between patients having less than 17 days and those having more than 17 days between the two MRI.

### 3.3 Comparison between phantom and patient features

Fifty-eight features (54 %) were considered as poorly robust on the whole study: 18 in common between phantom and patient data, 9 on the phantom study and 31 on the patient study. These common poorly robust features are outlined in Table 3.

**Table 3:** List of common poorly robust features.

Feature
original_firstorder_Variance
original_glcm_Autocorrelation
original_glcm_ClusterProminence
original_glcm_ClusterTendency
original_glcm_SumSquares
original_gldm_GrayLevelVariance
original_gldm_HighGrayLevelEmphasis
original_gldm_SmallDependenceHighGrayLevelEmphasis
original_glrlm_HighGrayLevelRunEmphasis
original_glrlm_ShortRunHighGrayLevelEmphasis
original_glszm_HighGrayLevelZoneEmphasis
original_glszm_LargeAreaEmphasis
original_glszm_LargeAreaLowGrayLevelEmphasis
original_glszm_SmallAreaHighGrayLevelEmphasis
original_glszm_ZoneVariance
original_ngtdm_Complexity
original_ngtdm_Strength
original_glcm_ClusterShade

## 4 Discussion

In this comprehensive study, we assessed the robustness of radiomic features captured by a 0.35 T MRI scanner integrated within the MRIdian® system, using a standard protocol for pancreatic cancer. The analysis focused on the reproducibility of these features extracted from two distinct sets of images: those obtained from a dedicated phantom and those from patients undergoing treatment. The study objective was to determine the repeatability of various radiomic features, providing a filter to remove those lacking robustness.

Dimensionality reduction is a critical phase in the process of radiomic analysis, aiming toreduce the risk of overfitting in predictive modeling. Overfitting can lead to models that perform exceptionally on training data but fail to generalize to new, unseen data. While machine and deep learning algorithms are commonly employed to perform this reduction, they may not necessarily exclude features that are non-robust under the conditions of acquisition, which is a significant consideration for ensuring the reliability of radiomic studies [12].

Numerous studies have illuminated the profound impact that even minor variations in MRI acquisition parameters can have on the values of radiomic features [13], [14], [15], [16]. Additionally, variations have been noted when identical sequences are deployed on systems from different manufacturers [17], [18], [19]. While post-processing steps have been shown to mitigate some of this variability, they fall short of establishing robustness across all radiomic features [20]. This underscores the necessity of test–retest studies that can identify and exclude features whose variability is attributed not to pathological or therapeutic changes but to inconsistencies in the imaging acquisition parameters themselves [21], [22].

In our analyses, we determined that 54 % of the radiomic features we extracted could be deemed non-reproducible and thus unsuitable for inclusion in subsequent analyses. The approach of dimensionality reduction used in this study is grounded in transparent methodologies, providing an explainable selection of features. This is in stark contrast to the usual opaque nature of artificial intelligence, particularly with deep learning algorithms, where the internal mechanics of feature selection are not always accessible or interpretable.

In our phantom studies, we identified 27 features that were not reproducible, and of these, a significant number were also found to be non-reproducible in the patient image analyses, suggesting a consistency in the feature behavior across different data sets. It was noted that most of the commonly non-reproducible features pertained to texture.. Interestingly, most first-order features that exhibited non-reproducibility in patient data analysis demonstrated good repeatability in phantom data analysis, highlighting a potential discrepancy between phantom and clinical scenarios.

There is a sparse yet growing body of literature exploring the robustness of radiomics features within systems of MR-guided radiotherapy like the MRIdian®.. Our findings align with some of these prior studies, particularly with regard to the robustness of shape-based features and specific textural features [23], [24]. Some of the robust features of the study of Ericsson-Szecsenyi *et al*. were also robust in our study (for example shape-based features, GLCM sum entropy, GLRLM short-run emphasis, GLRLM long-run emphasis, GLRLM run percentage, GLRLM run length non-uniformity) [23].

The application of Stereotactic Adaptive MR-guided Radiotherapy (SMART) for inoperable pancreatic cancers holds substantial promise, demonstrated by the emerging clinical results [25], [26]. However, with the reality that many patients still face the prospect of recurrence, the development of predictive tools is paramount. Various research groups have ventured to develop and propose radiomic-based models with the intent of predicting patient outcomes after SMART.t. Notably, some of these predictive models have achieved promising levels of accuracy, as evidenced by their reported Area Under the Curve (AUC) statistics [27], [28]. However, a common gap in these studies is the absence of a thorough reproducibility analysis of the radiomic features used within these predictive models.

The method of feature selection delineated in our study aims to ensure that only reproducible features are carried forward into the final radiomic models constructed by various machine learning algorithms.

While our study brings to light several important findings, it is not without its limitations. A notable one is the absence of an inter-observer segmentation analysis, a factor known to introduce variability in the delineation of GTV. By not addressing this variability, we acknowledge a potential source of error that could influence the reproducibility of the radiomic features.

Additionally, our investigation was conducted in a single-center setting, utilizing a singular MRIdian® system and a single imaging protocol with the same coil, acquisition parameters and resolution. The results would be different if any of the imaging parameters were different (i.e. coil, field of view, TE/TR, etc.). This would require a new test–retest study in order to select robust features. The results may also differ when applied to other systems or within different institutional protocols. To enhance the applicability of our findings, a multicentric approach involving various MRIdian® systems, preferably across different geographic locations and patient populations, would be instrumental. Such an approach would allow for the comparison of radiomic feature robustness in a broader context, potentially validating the findings and ensuring that the models developed are more universally applicable. Moreover, the evolving field of radiomics in radiotherapy demands ongoing dialogue between technological advancement and clinical application. As such, the continuous integration of newer imaging technologies and updated radiotherapy techniques will necessitate constant re-evaluation of radiomic feature robustness.

In conclusion, the scope of this study serves not only to refine the process of feature selection in radiomics but also to underscore the importance of rigorous validation in the field of medical imaging and oncology. By advancing methodologies that prioritize the reproducibility and reliability of data, we set a precedent for future research that seeks to harness the power of radiomics in the pursuit of personalized medicine.

## CRediT authorship contribution statement

**Morgan Michalet:** Conceptualization, Data curation, Investigation, Methodology, Writing – original draft, Writing – review & editing. **Gladis Valenzuela:** Investigation, Methodology. **Pierre Debuire:** Data curation. **Olivier Riou:** Writing – review & editing. **David Azria:** Writing – review & editing. **Stéphanie Nougaret:** Writing – review & editing. **Marion Tardieu:** Conceptualization, Data curation, Investigation, Methodology, Writing – original draft, Writing – review & editing.

## Declaration of competing interest

The authors declare that they have no known competing financial interests or personal relationships that could have appeared to influence the work reported in this paper.
