# Supplementary material for: Robustness of radiomics features on 0.35 T magnetic resonance imaging for magnetic resonance-guided radiotherapy
Source: Phys Imaging Radiat Oncol. 2024 Jul 20;31:100613. doi: 10.1016/j.phro.2024.100613 (PMC11320460; doi:10.1016/j.phro.2024.100613)
Supplement: Supplementary Data 1 [file mmc1.docx]

**Table S1: Extracted features for phantom images analysis**

| 'original_firstorder_10Percentile' |
| --- |
| 'original_firstorder_90Percentile' |
| 'original_firstorder_Energy' |
| 'original_firstorder_Entropy' |
| 'original_firstorder_InterquartileRange' |
| 'original_firstorder_Kurtosis' |
| 'original_firstorder_Maximum' |
| 'original_firstorder_MeanAbsoluteDeviation' |
| 'original_firstorder_Mean' |
| 'original_firstorder_Median' |
| 'original_firstorder_Minimum' |
| 'original_firstorder_Range' |
| 'original_firstorder_RobustMeanAbsoluteDeviation' |
| 'original_firstorder_RootMeanSquared' |
| 'original_firstorder_Skewness' |
| 'original_firstorder_TotalEnergy' |
| 'original_firstorder_Uniformity' |
| 'original_firstorder_Variance' |
| 'original_glcm_Autocorrelation' |
| 'original_glcm_ClusterProminence' |
| 'original_glcm_ClusterShade' |
| 'original_glcm_ClusterTendency' |
| 'original_glcm_Contrast' |
| 'original_glcm_Correlation' |
| 'original_glcm_DifferenceAverage' |
| 'original_glcm_DifferenceEntropy' |
| 'original_glcm_DifferenceVariance' |
| 'original_glcm_Id' |
| 'original_glcm_Idm' |
| 'original_glcm_Idmn' |
| 'original_glcm_Idn' |
| 'original_glcm_Imc1' |
| 'original_glcm_Imc2' |
| 'original_glcm_InverseVariance' |
| 'original_glcm_JointAverage' |
| 'original_glcm_JointEnergy' |
| 'original_glcm_JointEntropy' |
| 'original_glcm_MCC' |
| 'original_glcm_MaximumProbability' |
| 'original_glcm_SumAverage' |
| 'original_glcm_SumEntropy' |
| 'original_glcm_SumSquares' |
| 'original_gldm_DependenceEntropy' |
| 'original_gldm_DependenceNonUniformity' |
| 'original_gldm_DependenceNonUniformityNormalized' |
| 'original_gldm_DependenceVariance' |
| 'original_gldm_GrayLevelNonUniformity' |
| 'original_gldm_GrayLevelVariance' |
| 'original_gldm_HighGrayLevelEmphasis' |
| 'original_gldm_LargeDependenceEmphasis' |
| 'original_gldm_LargeDependenceHighGrayLevelEmphasis' |
| 'original_gldm_LargeDependenceLowGrayLevelEmphasis' |
| 'original_gldm_LowGrayLevelEmphasis' |
| 'original_gldm_SmallDependenceEmphasis' |
| 'original_gldm_SmallDependenceHighGrayLevelEmphasis' |
| 'original_gldm_SmallDependenceLowGrayLevelEmphasis' |
| 'original_glrlm_GrayLevelNonUniformity' |
| 'original_glrlm_GrayLevelNonUniformityNormalized' |
| 'original_glrlm_GrayLevelVariance' |
| 'original_glrlm_HighGrayLevelRunEmphasis' |
| 'original_glrlm_LongRunEmphasis' |
| 'original_glrlm_LongRunHighGrayLevelEmphasis' |
| 'original_glrlm_LongRunLowGrayLevelEmphasis' |
| 'original_glrlm_LowGrayLevelRunEmphasis' |
| 'original_glrlm_RunEntropy' |
| 'original_glrlm_RunLengthNonUniformity' |
| 'original_glrlm_RunLengthNonUniformityNormalized' |
| 'original_glrlm_RunPercentage' |
| 'original_glrlm_RunVariance' |
| 'original_glrlm_ShortRunEmphasis' |
| 'original_glrlm_ShortRunHighGrayLevelEmphasis' |
| 'original_glrlm_ShortRunLowGrayLevelEmphasis' |
| 'original_glszm_GrayLevelNonUniformity' |
| 'original_glszm_GrayLevelNonUniformityNormalized' |
| 'original_glszm_GrayLevelVariance' |
| 'original_glszm_HighGrayLevelZoneEmphasis' |
| 'original_glszm_LargeAreaEmphasis' |
| 'original_glszm_LargeAreaHighGrayLevelEmphasis' |
| 'original_glszm_LargeAreaLowGrayLevelEmphasis' |
| 'original_glszm_LowGrayLevelZoneEmphasis' |
| 'original_glszm_SizeZoneNonUniformity' |
| 'original_glszm_SizeZoneNonUniformityNormalized' |
| 'original_glszm_SmallAreaEmphasis' |
| 'original_glszm_SmallAreaHighGrayLevelEmphasis' |
| 'original_glszm_SmallAreaLowGrayLevelEmphasis' |
| 'original_glszm_ZoneEntropy' |
| 'original_glszm_ZonePercentage' |
| 'original_glszm_ZoneVariance' |
| 'original_ngtdm_Busyness' |
| 'original_ngtdm_Coarseness' |
| 'original_ngtdm_Complexity' |
| 'original_ngtdm_Contrast' |
| 'original_ngtdm_Strength' |

**Table S2: List of highly repeatable features on phantom images and their coefficient of variation (CoV < 5 %)**

| Feature | Coefficient of variation (CoV) |
| --- | --- |
| original_firstorder_Entropy | 1.6% |
| original_firstorder_InterquartileRange | 4.5% |
| original_firstorder_Kurtosis | 4.7% |
| original_firstorder_Mean | 4.8% |
| original_firstorder_Median | 4.8% |
| original_firstorder_RootMeanSquared | 4.8% |
| original_firstorder_Skewness | 2.5% |
| original_firstorder_Uniformity | 4.4% |
| original_glcm_Correlation | 2.8% |
| original_glcm_DifferenceAverage | 4.7% |
| original_glcm_DifferenceEntropy | 1.7% |
| original_glcm_Id | 1.7% |
| original_glcm_Idm | 2.2% |
| original_glcm_Idmn | 0.0% |
| original_glcm_Idn | 0.0% |
| original_glcm_Imc1 | 3.6% |
| original_glcm_Imc2 | 1.1% |
| original_glcm_InverseVariance | 1.6% |
| original_glcm_JointEntropy | 1.7% |
| original_glcm_MCC | 2.1% |
| original_glcm_SumEntropy | 1.4% |
| original_gldm_DependenceEntropy | 0.6% |
| original_gldm_DependenceNonUniformity | 2.8% |
| original_gldm_DependenceNonUniformityNormalized | 2.7% |
| original_gldm_DependenceVariance | 3.5% |
| original_gldm_GrayLevelNonUniformity | 4.2% |
| original_gldm_LargeDependenceEmphasis | 4.7% |
| original_gldm_SmallDependenceEmphasis | 4.2% |
| original_glrlm_GrayLevelNonUniformity | 2.9% |
| original_glrlm_GrayLevelNonUniformityNormalized | 3.9% |
| original_glrlm_LongRunEmphasis | 2.5% |
| original_glrlm_RunEntropy | 0.8% |
| original_glrlm_RunLengthNonUniformity | 2.1% |
| original_glrlm_RunLengthNonUniformityNormalized | 1.2% |
| original_glrlm_RunPercentage | 0.8% |
| original_glrlm_ShortRunEmphasis | 0.5% |
| original_glszm_GrayLevelNonUniformity | 1.9% |
| original_glszm_GrayLevelNonUniformityNormalized | 3.1% |
| original_glszm_LargeAreaHighGrayLevelEmphasis | 3.1% |
| original_glszm_SizeZoneNonUniformityNormalized | 1.1% |
| original_glszm_SmallAreaEmphasis | 0.6% |
| original_glszm_ZoneEntropy | 0.5% |
| original_glszm_ZonePercentage | 4.6% |
| original_ngtdm_Coarseness | 1.6% |

CoV = Coefficient of variation

**Table S3: List of repeatable features on phantom images and their coefficient of variation (5% < CoV < 10 %)**

| Feature | Coefficient of variation (CoV) |
| --- | --- |
| original_firstorder_10Percentile | 5.4% |
| original_firstorder_Energy | 9.7% |
| original_firstorder_Maximum | 5.4% |
| original_firstorder_MeanAbsoluteDeviation | 5.8% |
| original_firstorder_Range | 5.8% |
| original_firstorder_RobustMeanAbsoluteDeviation | 8.2% |
| original_firstorder_TotalEnergy | 9.7% |
| original_glcm_Contrast | 9.5% |
| original_glcm_DifferenceVariance | 9.5% |
| original_glcm_JointAverage | 5.4% |
| original_glcm_JointEnergy | 7.9% |
| original_glcm_MaximumProbability | 7.0% |
| original_glcm_SumAverage | 5.4% |
| original_gldm_LargeDependenceHighGrayLevelEmphasis | 5.9% |
| original_glrlm_GrayLevelVariance | 9.6% |
| original_glrlm_LongRunHighGrayLevelEmphasis | 8.2% |
| original_glrlm_RunVariance | 5.1% |
| original_glszm_GrayLevelVariance | 9.1% |
| original_glszm_SizeZoneNonUniformity | 5.7% |
| original_ngtdm_Busyness | 9.9% |
| original_ngtdm_Contrast | 6.7% |

CoV = Coefficient of variation

**Table S4: Extracted features for patient images analysis**

| original_shape_SurfaceArea |
| --- |
| original_firstorder_RootMeanSquared |
| original_shape_SurfaceVolumeRatio |
| original_shape_Maximum2DDiameterColumn |
| original_glcm_Imc2 |
| original_glcm_JointAverage |
| original_glcm_Contrast |
| original_glrlm_GrayLevelNonUniformity |
| original_gldm_HighGrayLevelEmphasis |
| original_shape_Maximum2DDiameterSlice |
| original_glcm_JointEnergy |
| original_glcm_Imc1 |
| original_firstorder_Range |
| original_gldm_DependenceNonUniformityNormalized |
| original_shape_Maximum3DDiameter |
| original_glszm_LargeAreaHighGrayLevelEmphasis |
| original_glszm_ZoneEntropy |
| original_glszm_LargeAreaLowGrayLevelEmphasis |
| original_glcm_Id |
| original_glcm_DifferenceEntropy |
| original_glcm_MaximumProbability |
| original_shape_Sphericity |
| original_gldm_LargeDependenceHighGrayLevelEmphasis |
| original_ngtdm_Strength |
| original_gldm_LowGrayLevelEmphasis |
| original_glszm_GrayLevelVariance |
| original_shape_MeshVolume |
| original_glszm_SmallAreaLowGrayLevelEmphasis |
| original_glcm_SumAverage |
| original_glszm_ZoneVariance |
| original_shape_LeastAxisLength |
| original_firstorder_InterquartileRange |
| original_firstorder_Maximum |
| original_firstorder_90Percentile |
| original_firstorder_10Percentile |
| original_glcm_Correlation |
| original_firstorder_Variance |
| original_glcm_ClusterTendency |
| original_glrlm_RunLengthNonUniformity |
| original_glszm_SmallAreaHighGrayLevelEmphasis |
| original_glcm_DifferenceVariance |
| original_glrlm_ShortRunLowGrayLevelEmphasis |
| original_glcm_MCC |
| original_glszm_LargeAreaEmphasis |
| original_gldm_LargeDependenceEmphasis |
| original_gldm_DependenceNonUniformity |
| original_glszm_SmallAreaEmphasis |
| original_gldm_DependenceVariance |
| original_glcm_SumSquares |
| original_shape_Maximum2DDiameterRow |
| original_firstorder_Skewness |
| original_glszm_ZonePercentage |
| original_glrlm_ShortRunEmphasis |
| original_glrlm_GrayLevelVariance |
| original_glrlm_ShortRunHighGrayLevelEmphasis |
| original_gldm_LargeDependenceLowGrayLevelEmphasis |
| original_glrlm_LongRunEmphasis |
| original_firstorder_Entropy |
| original_gldm_SmallDependenceLowGrayLevelEmphasis |
| original_glrlm_RunLengthNonUniformityNormalized |
| original_glcm_SumEntropy |
| original_firstorder_Kurtosis |
| original_firstorder_RobustMeanAbsoluteDeviation |
| original_firstorder_Mean |
| original_firstorder_Energy |
| original_firstorder_Median |
| original_gldm_SmallDependenceHighGrayLevelEmphasis |
| original_glcm_Idmn |
| original_glrlm_LongRunLowGrayLevelEmphasis |
| original_glcm_InverseVariance |
| original_glszm_HighGrayLevelZoneEmphasis |
| original_glrlm_RunEntropy |
| original_shape_MinorAxisLength |
| original_firstorder_Minimum |
| original_glrlm_LowGrayLevelRunEmphasis |
| original_ngtdm_Busyness |
| original_glrlm_GrayLevelNonUniformityNormalized |
| original_ngtdm_Coarseness |
| original_firstorder_Uniformity |
| original_glszm_GrayLevelNonUniformity |
| original_glrlm_HighGrayLevelRunEmphasis |
| original_gldm_GrayLevelVariance |
| original_shape_VoxelVolume |
| original_glcm_Autocorrelation |
| original_ngtdm_Contrast |
| original_glrlm_RunVariance |
| original_shape_Flatness |
| original_shape_Elongation |
| original_gldm_SmallDependenceEmphasis |
| original_glszm_GrayLevelNonUniformityNormalized |
| original_glcm_Idn |
| original_ngtdm_Complexity |
| original_glszm_LowGrayLevelZoneEmphasis |
| original_gldm_DependenceEntropy |
| original_glcm_ClusterProminence |
| original_glcm_JointEntropy |
| original_firstorder_MeanAbsoluteDeviation |
| original_glcm_Idm |
| original_glcm_ClusterShade |
| original_glrlm_LongRunHighGrayLevelEmphasis |
| original_glcm_DifferenceAverage |
| original_glszm_SizeZoneNonUniformity |
| original_glszm_SizeZoneNonUniformityNormalized |
| original_gldm_GrayLevelNonUniformity |
| original_firstorder_TotalEnergy |
| original_shape_MajorAxisLength |
| original_glrlm_RunPercentage |

**Table S5: List of highly-reproducible GTV features on simulation vs first fraction images and their interclass correlation (ICC > 90%)**

| Feature | Interclass Correlation (ICC) |
| --- | --- |
| original_shape_SurfaceArea | 96.4% |
| original_shape_SurfaceVolumeRatio | 98.5% |
| original_shape_Maximum2DDiameterColumn | 98.5% |
| original_glrlm_GrayLevelNonUniformity | 91.6% |
| original_shape_Maximum2DDiameterSlice | 97.4% |
| original_glcm_JointEnergy | 95.6% |
| original_shape_Maximum3DDiameter | 97.5% |
| original_glszm_ZoneEntropy | 92.6% |
| original_shape_Sphericity | 96.7% |
| original_shape_MeshVolume | 92.7% |
| original_glszm_SmallAreaLowGrayLevelEmphasis | 96.0% |
| original_shape_LeastAxisLength | 95.4% |
| original_glrlm_RunLengthNonUniformity | 91.5% |
| original_shape_Maximum2DDiameterRow | 96.2% |
| original_gldm_SmallDependenceLowGrayLevelEmphasis | 93.7% |
| original_glcm_Idmn | 90.7% |
| original_shape_MinorAxisLength | 97.2% |
| original_glrlm_GrayLevelNonUniformityNormalized | 93.3% |
| original_ngtdm_Coarseness | 95.1% |
| original_firstorder_Uniformity | 93.2% |
| original_shape_VoxelVolume | 92.7% |
| original_shape_Flatness | 97.1% |
| original_shape_Elongation | 95.9% |
| original_glszm_GrayLevelNonUniformityNormalized | 92.0% |
| original_glszm_LowGrayLevelZoneEmphasis | 93.4% |
| original_gldm_DependenceEntropy | 92.2% |
| original_gldm_GrayLevelNonUniformity | 91.1% |
| original_shape_MajorAxisLength | 98.1% |

ICC = Interclass correlation

**Table S6: List of reproducible GTV features on simulation vs first fraction images and their interclass correlation (90% > ICC > 75%)**

| Feature | Interclass Correlation (ICC) |
| --- | --- |
| original_glcm_Imc2 | 82.6% |
| original_glcm_Imc1 | 80.2% |
| original_glszm_LargeAreaHighGrayLevelEmphasis | 89.9% |
| original_glcm_Id | 75.5% |
| original_glcm_MaximumProbability | 87.9% |
| original_gldm_LowGrayLevelEmphasis | 86.1% |
| original_glcm_Correlation | 86.6% |
| original_glrlm_ShortRunLowGrayLevelEmphasis | 86.9% |
| original_glcm_MCC | 76.6% |
| original_gldm_LargeDependenceEmphasis | 76.5% |
| original_gldm_DependenceNonUniformity | 82.7% |
| original_glrlm_ShortRunEmphasis | 79.3% |
| original_gldm_LargeDependenceLowGrayLevelEmphasis | 85.3% |
| original_glrlm_LongRunEmphasis | 77.7% |
| original_firstorder_Entropy | 75.8% |
| original_glrlm_RunLengthNonUniformityNormalized | 78.7% |
| original_glcm_SumEntropy | 79.5% |
| original_firstorder_Kurtosis | 80.6% |
| original_glrlm_LongRunLowGrayLevelEmphasis | 87.3% |
| original_glcm_InverseVariance | 76.8% |
| original_glrlm_RunEntropy | 75.2% |
| original_firstorder_Minimum | 86.8% |
| original_glrlm_LowGrayLevelRunEmphasis | 86.8% |
| original_ngtdm_Busyness | 80.7% |
| original_glszm_GrayLevelNonUniformity | 89.4% |
| original_glrlm_RunVariance | 75.4% |
| original_glcm_Idn | 83.5% |
| original_glcm_JointEntropy | 83.9% |
| original_glcm_Idm | 77.8% |
| original_glrlm_RunPercentage | 78.0% |

ICC = Interclass correlation

**Table S7: Influence of time (> or ≤ 17 days) between the simulation MRI and the first fraction MRI on the variation of 4 low-reproducible (ICC < 0.75) and 4 high-reproducible (ICC > 0.75) features**

| Feature | ICC | Mean (+/- std) variation between simulation and Fr1 (5-17 days) | Mean (+/- std) variation between simulation and Fr1 (18-33 days) | Equivalence Test P-value * |
| --- | --- | --- | --- | --- |
| original_glszm_SmallAreaHighGrayLevelEmphasis | 0.077 | 0.23 (0.28) | 0.28 (0.32) | 0.082 |
| original_glcm_SumAverage | 0.279 | 0.14 (0.18) | 0.14 (0.21) | 0.001 |
| original_firstorder_10Percentile | 0.399 | 0.14 (0.17) | 0.15 (0.20) | 0.001 |
| original_gldm_DependenceNonUniformityNormalized | 0.609 | 0.13 (0.12) | 0.13 (0.14) | <0.001 |
| original_glrlm_LongRunEmphasis | 0.777 | 0.05 (0.04) | 0.05 (0.04) | 0.004 |
| original_glcm_Correlation | 0.866 | 0.05 (0.04) | 0.04 (0.07) | 0.025 |
| original_gldm_GrayLevelNonUniformity | 0.911 | 0.15 (0.15) | 0.20 (0.31) | 0.040 |
| original_glszm_SmallAreaLowGrayLevelEmphasis | 0.96 | 0.30 (0.22) | 0.27 (0.22) | 0.009 |

ICC = Interclass correlation; Fr1 = first treatment fraction

* Equivalence limit considered: 0.15 for features with a std > 0.10 and 0.03 for features with a std <0.10.


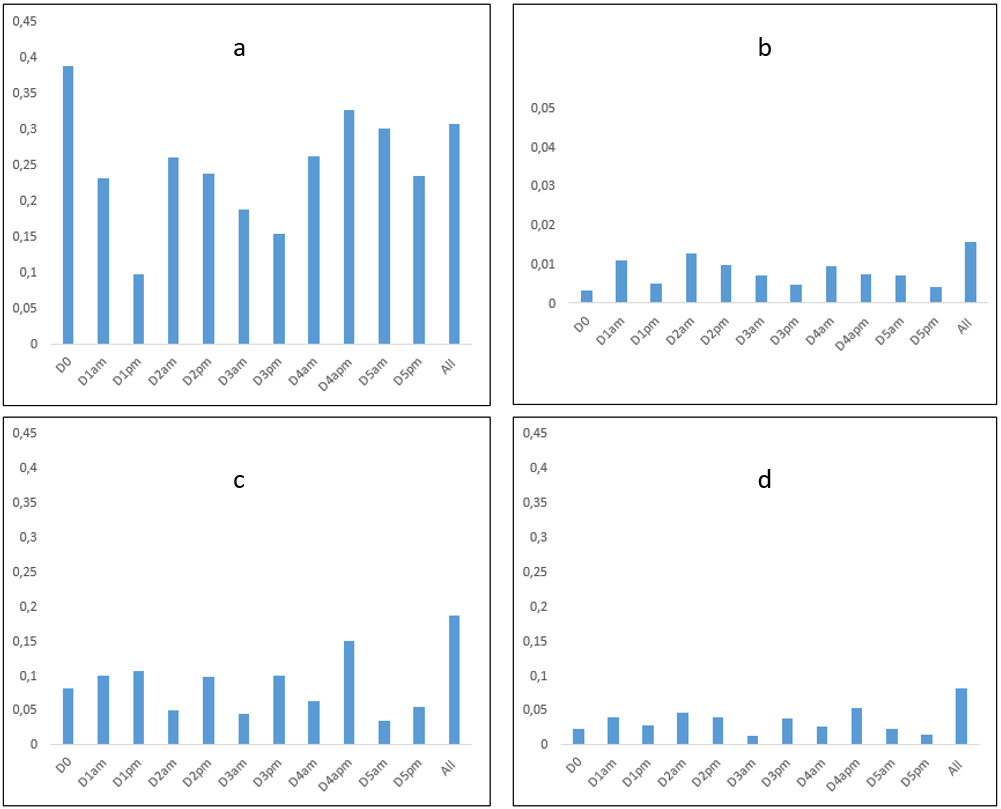


*Figure S1: example of CoV variations for 4 different features (a- first order feature “minimum”, b- first order feature “entropy”, c- texture feature “LRLGL emphasis”, d- texture feature “LRGL emphasis”) , D = day, am = ante meridian, pm= post meridiem, CoV = coefficient of variation*
